# Maternal low glycaemic index diet, fat intake and postprandial glucose influences neonatal adiposity – secondary analysis from the ROLO study

**DOI:** 10.1186/1475-2891-13-78

**Published:** 2014-08-01

**Authors:** Mary K Horan, Ciara A McGowan, Eileen R Gibney, Jean M Donnelly, Fionnuala M McAuliffe

**Affiliations:** 1 UCD Obstetrics and Gynaecology, School of Medicine and Medical Science, University College Dublin, National Maternity Hospital, Dublin 2, Ireland; 2 Science Centre – South, University College Dublin School Of Agriculture & Food Science, Belfield, Dublin 4, Ireland

**Keywords:** Maternal diet, Pregnancy, Neonatal body composition, Neonatal adiposity, Low glycaemic index diet

## Abstract

**Background:**

The in utero environment is known to affect fetal development however many of the mechanisms by which this occurs remain unknown. The aim of this study was to examine the association between maternal dietary macronutrient intake and lifestyle throughout pregnancy and neonatal weight and adiposity.

**Methods:**

This was an analysis of 542 mother and infant pairs from the ROLO study (Randomised cOntrol trial of LOw glycaemic index diet versus no dietary intervention to prevent recurrence of fetal macrosomia). Food diaries as well as food frequency and lifestyle and physical activity questionnaires were completed during pregnancy. Maternal anthropometry was measured throughout pregnancy and neonatal anthropometry was measured at birth.

**Results:**

Multiple linear regression analysis revealed the main maternal factor associated with increased birth weight was greater gestational weight gain R^2^_adj_23.3% (F = 11.547, p < 0.001). The main maternal factor associated with increased birth length was non-smoking status R^2^_adj_27.8% (F = 6.193, p < 0.001). Neonatal central adiposity (determined using waist:length ratio) was negatively associated with maternal age, and positively associated with the following parameters: smoking status, maternal pre-pregnancy arm circumference, percentage energy from saturated fat in late pregnancy, postprandial glucose at 28 weeks gestation and membership of the control group with a positive trend towards association with trimester 2 glycaemic load R^2^_adj_ 38.1% (F = 8.000, p < 0.001).

**Conclusions:**

Several maternal diet and lifestyle factors were associated with neonatal anthropometry . Low glycaemic index dietary intervention in pregnancy was found to have a beneficial effect on neonatal central adiposity. Additionally, central adiposity was positively associated with maternal dietary fat intake and postprandial glucose highlighting the important role of healthy diet in pregnancy in promoting normal neonatal adiposity.

**Trial registration:**

Current Controlled Trials ISRCTN54392969.

## Background

The in utero environment has been found to affect fetal development in a variety of ways from cognitive development [1] to development of the fetal organs [2] to growth and fat deposition [3]. Environmental factors to which the pregnant woman is exposed result in epigenetic changes which impact on fetal genetic transcription and affect the fetus differently depending on the stage of pregnancy [2,4,5]. The maternal diet during pregnancy is particularly important as the mother is relied on to provide all of the nutrients required for the fetus to grow and develop [6], however maternal pre-pregnancy nutritional status and lifestyle have also been found to be important [7]. Pre-pregnancy overweight and obesity as well as excessive gestational weight gain have been found to result in fetal macrosomia i.e. a birthweight of ≥4 kg [7]. Macrosomic infants are at increased risk of developing metabolic syndrome in later life and this effect may persist in later generations through intergenerational programming [8]. Macrosomia also increases obstetric risks such as shoulder dystocia, maternal anal sphincter injury, instrumental vaginal delivery and emergency caesarean section [9].

The hyperglycaemia-hyperinsulinism theory proposes that the mother’s intake of carbohydrate and her natural pregnancy-related progressive insulin insensitivity results in higher levels of maternal blood glucose which is transferred to the fetus [10]. In response, the fetus produces its own insulin which then acts as a growth hormone resulting in increased fetal growth and adiposity [9,10]. Studies support this hypothesis during gestational diabetes [11] and also within normal limits of maternal blood glucose [12]. As such it is important for maternal blood glucose levels to be maintained within normal levels to ensure fetal glucose levels, and subsequent insulin levels are also maintained within normal ranges.

Other dietary factors including maternal dietary energy and protein intake have also been found to influence neonatal anthropometry [13-15] while the quality of macronutrients also appears to have a role, in particular the quality of fat i.e. saturated or trans-fat vs polyunsaturated fat intake [16,17] and the type of protein i.e. dairy vs meat protein [18]. Maternal micronutrient intakes also affect neonatal body composition however even less data is available in this area except in the area of deficiency and supplementation in the developing world in order to reduce preterm birth and small for gestational age infants [19,20]. Therefore, this study focuses on the association between maternal macronutrient intake throughout pregnancy and neonatal body composition. The relationship between neonatal size and maternal dietary intake is not clear cut since, in addition to diet, a variety of maternal characteristics and lifestyle factors including physical activity, socioeconomic and demographic status, stress, smoking, alcohol consumption and other drug intake as well as genetic factors also affect fetal growth and development [4,21-23]. Due to the complex nature of the determinants of neonatal size and adiposity and the fact that the most commonly reported anthropometric parameters “weight” and “length” are very limited measures of adiposity which give no information on body fat distribution [24] there remains a paucity of data in the area. The aim of this study was to use a cohort from the ROLO (Randomised cOntrol trial of LOw glycaemic index diet versus no dietary intervention to prevent recurrence of fetal macrosomia) study to examine the effect of maternal body composition, demographic characteristics, macronutrient intake and lifestyle both pre-pregnancy and throughout pregnancy on neonatal weight and adiposity.

## Methods

542 mother and infant pairs from the ROLO study were included in this analysis. The ROLO study was a randomised control trial of 800 secundigravida women with a previous macrosomic baby (>4 kg) randomised to receive low glycaemic index (GI) dietary advice versus usual care (no dietary advice) to reduce recurrence of macrosomia. Detailed methodology and results of the ROLO study, which was carried out in the National Maternity Hospital, Ireland, have previously been published [25,26] but in brief; the primary outcome was birthweight and the secondary outcomes were gestational weight gain and glucose intolerance. Low GI dietary advice was given at week 14 of pregnancy while demographic, well-being and lifestyle questionnaires were returned by 28 weeks gestation. 3-day food diaries were also completed in each trimester of pregnancy and used to determine the glycaemic index and glycaemic load of the women’s diets. The control group received no dietary advice and had routine antenatal care. The ROLO study found that the intervention group significantly lowered their glycaemic index and glycaemic load and had significantly lower gestational weight gain and glucose intolerance but birthweight or risk of macrosomia was not significantly reduced [25,27]. This study was conducted according to the guidelines laid down in the Declaration of Helsinki with ethics approval from the National Maternity Hospital review board and written informed maternal consent.

### Inclusion and exclusion criteria

Participants were secundigravida women with a previous macrosomic baby (>4 kg). They were required to have sufficient literacy and English language fluency to understand the intervention and be capable of completing questionnaires. Women were only included if they had healthy, singleton pregnancies with no intrauterine growth abnormalities.

### Maternal demographics, lifestyle and well-being

Of the 800 participants of the ROLO study, 542 completed questionnaires in the first half of pregnancy exploring various background socioeconomic and socio-demographic, and lifestyle variables. Questions from SLAN (Survey of Lifestyle, Attitudes and Nutrition in Ireland) [28] relating to lifestyle habits were completed, including questions on physical activity, smoking and educational attainment. Well-being was measured using the World Health Organisation 5-Item Index (WHO-5) expressed as a percentage score from 0-100% i.e. the lowest to highest possible wellbeing scores [29].

### Maternal and neonatal anthropometry

Maternal weight, height and mid-upper arm circumference were measured at the first antenatal consultation and BMI calculated. Maternal weight was also measured at each subsequent consultation and gestational weight gain calculated.

Neonatal weight, length, mid-upper arm, abdominal, hip and thigh circumference, and biceps, triceps, subscapular and thigh skinfold measurements were taken at birth. Weight and length were measured for all 542 neonates while other anthropometric measurements began to be taken later in the study and therefore complete data were available for 266 neonates. Waist:hip, waist:length and triceps skinfold:subscapular skinfold ratios as well as sum of triceps and subscapular skinfold thicknesses and sum of all skinfold thicknesses were calculated as a measure of neonatal adiposity.

### Maternal dietary intake

3-day food diaries were completed in each trimester of pregnancy and used to determine macronutrient intake as well as the glycaemic index and glycaemic load of the women’s diet. Macronutrients were expressed as a percentage of total energy. Macronutrient intake during each trimester of pregnancy was examined separately. Underreporting was examined using Goldberg ratios ie the ratio of energy intake to estimated basal metabolic rate [30]. Basal metabolic rate was calculated using Schofield equations and a Goldberg ratio of ≤0.9 was used to identify definite underreporters [30-32].

Cluster analysis had been previously completed for the control group of this cohort using 3 day food diaries to define food group intake at each trimester which was then analysed using k-means cluster analysis resulting in the identification of 2 main clusters of “healthy” and “unhealthy” individuals with regard to diet [33]. In brief the unhealthy dietary cluster was characterised by significantly higher intakes of white bread, refined breakfast cereals, confectionery, chips, processed meats and high-energy beverages. The second cluster was characterised by significantly higher intakes of wholegrain breads and breakfast cereals, fruit, vegetables, fruit juice, fish, low-fat milk and white meat [33]. Dietary intake over the past 3 months was examined using the self-administered 170 item SLAN (Survey of Lifestyle, Attitudes and Nutrition in Ireland) food frequency questionnaire (FFQ) which was given in early pregnancy and returned by 28 weeks gestation. The SLAN FFQ was originally adapted from the European Prospective Investigation of Cancer (EPIC) study and has been validated for use in the Irish adult population [34,35]. Dietary data from the completed FFQs was used to create a Dietary Approaches to Stop Hypertension (DASH) index i.e. a score measuring the level of concordance with the DASH diet from 0 which indicates total non-concordance to 11 which indicates total concordance [36]. The DASH diet was developed for the reduction of hypertension but, as with other dietary indices, also indicates overall healthy diet [37].

Full methodology for entry and analysis of the dietary intake of participants has previously been published [27]. In brief, all food diaries and food frequency questionnaires were entered by a trained dietitian/clinical nutritionist with the use of the household measures and UK Food Standards Agency average portion sizes [38]. Food Diaries were analysed using Tinuviel WISP software, version 3.0, in which the food composition tables used are derived from the 6th edition of McCance and Widdowson’s Food Composition Tables [39]. Food Frequency Questionnaires were analysed using Tinuviel QBuilder software, version 2.0, which also uses food compositions from the 6th edition of McCance and Widdowson’s Food Composition Tables [39]. GI values were updated in WISP and QBuilder from 2002 values using the 2008 International Tables of Glycaemic Index Values and other more recently published GI values [40,41].

### Oral glucose challenge test (GCT)

At 28 weeks gestation, fasting glucose was measured and a glucose challenge test (GCT) measuring serum glucose one hour post 50 g glucose load was performed. Postprandial blood glucose levels were recorded with results ≥7.8 mmol/L categorised as glucose intolerance [42,43]. GCT is normally carried out as part of institutional policy only if women have specific risk factors for gestational diabetes. If women have a blood glucose level of > 8.3 mmol/L 1 hour post GCT, formal glucose tolerance testing is carried out on a different day to rule out gestational diabetes. However, all participants of the ROLO study underwent GCT as part of the study protocol. They were then referred for glucose tolerance testing only if blood glucose level was > 8.3 mmol/L in accordance with institutional policy.

### Statistical analysis

Statistical analysis was completed using SPSS (Statistical Package for the Social Sciences) software version 20.0. Statistical analyses involved correlations, independent sample t-tests, ANOVA, ANCOVA, simple and multiple linear regression modelling. The intervention and control groups were analysed both separately and together to ensure all results were representative of both groups. Because there was no difference in neonatal anthropometry except for thigh circumference [44] and waist:length ratio between the control and intervention groups, groups were not analysed separately for final analysis but group was controlled for in all final models. Analysis was also carried out both including and excluding definite underreporters of dietary intake and since the exclusion of these underreporters did not change any of the significant associations but did decrease the power of the analysis, dietary underreporters were not excluded when presenting final models with the exception of the waist:length circumference whose associations were affected. In order to determine whether the loss of significance of some associations in this model was due to a loss of power or actually due to the effect of underreporting, underreporting was then controlled for in this model and both the adjusted and unadjusted models presented. Healthiness of the diet was compared to neonatal anthropometry by examining dietary clusters (as defined above) and the DASH dietary index using simple linear regression. In order to build multiple regression models, variables (including macronutrient intake for each trimester, parental height, weight and BMI, gestational weight gain, maternal physical activity, smoking status, ethnicity, age, marital/partner status, oral glucose challenge test results, alcohol intake and glycaemic index and load status) were first analysed using correlations, independent sample t-tests and ANOVA as appropriate. Variables that were found to be significantly associated with neonatal anthropometry were further analysed using simple linear regression then imputed into the final multiple regression model for well-being using a forced enter and backwards stepwise approach. Variables that were statistically significantly associated with neonatal anthropometry using simple linear regression were then included in a backwards stepwise multiple regression block resulting in any non-significant variables being discarded from the model in a stepwise manner. Variables known to effect neonatal size (education level as a marker of socioeconomic status, maternal pre-pregnancy BMI, length of gestation and neonate gender) were controlled for using a forced enter multiple regression block in all models. As mentioned, membership of the control or intervention group was also included by forced enter multiple regression in these models. Multiple linear regression resulted in a best and final model and those that were statistically significant overall (p < 0.05) were chosen as those which best predicted neonatal anthropometric measurements.

## Results

### Demographics, lifestyle and well-being

Maternal characteristics are listed in Table 1. Maternal characteristics did not differ between the control and intervention groups except for gestational weight gain [25,27], glucose intolerance and maternal well-being score [29] as previously described. 91.4% of the women were of “white Irish”, 6.7% of “white other”, 0.3% of “African”, 0.5% of “Chinese”, 0.1% of “Indian” and 1.0% of “Filipino/South East Asian” ethnicity. Again, there was no difference in ethnicity between the control and intervention groups (p = 0.159). 78.1% of the women had achieved 3rd level education while 21.9% had not and there was no difference in these rates between the control and intervention groups (p = 0.680), similarly there was no difference between reported smoking status between women in the control and intervention groups with 4.0% smokers and 96.0% non-smokers (p = 0.208). There was also no difference in physical activity levels between the control and intervention groups at baseline.

**Table 1:** General maternal characteristics during pregnancy, neonatal anthropometry and comparison of control and intervention (low glycaemic index diet) groups

	**n**	**Intervention**	**Control**	**Total**	**p-value**
Mother Age (yrs)	542	32.83 ± 3.97	32.91 ± 3.91	32.87 ± 3.93	0.824
Mother Height (cm)	542	165.58 ± 12.29	165.03 ± 11.54	165.27 ± 11.86	0.591
Mother Weight (kg)	542	72.45 ± 12.95	72.32 ± 12.97	72.38 ± 12.94	0.905
Mother BMI^1^ (kg/m^2^)	542	26.19 ± 4.35	26.38 ± 4.42	26.30 ± 4.38	0.616
Gestational weight gain (kg)	273	12.48 ± 4.40	14.13 ± 4.55	13.39 ± 4.55	0.003
Postprandial glucose*	537	6.47 ± 1.42	6.77 ± 1.77	6.64 ± 1.63	0.031
Mother well-being% score	508	56.37 ± 15.38	60.21 ± 15.19	58.48 ± 15.37	0.005
Days per week walking ≥30 min^2^	426	3.55 ± 1.86	3.44 ± 1.74	3.48 ± 1.79	0.527
Moderate activity (min per week)	308	70.80 ± 49.70	61.80 ± 36.64	66.20 ± 43.46	0.066
T1 Energy Intake (kcal/d)	521	1828.25 ± 407.98	1874.02 ± 474.10	1854.47 ± 446.12	0.245
T2 Energy Intake (kcal/d)	529	1803.43 ± 440.82	1943.24 ± 476.75	1883.50 ± 467.54	0.001
T3 Energy Intake (kcal/d)	541	1832.55 ± 424.02	1932.42 ± 472.56	1889.62 ± 454.91	0.011
T1 Protein Intake (%TE)	521	17.23 ± 3.04	16.80 ± 3.10	16.98 ± 3.08	0.114
T2 Protein Intake (%TE)	529	17.79 ± 3.16	16.75 ± 2.92	17.21 ± 3.07	<0.001
T3 Protein Intake (%TE)	541	17.62 ± 3.18	16.69 ± 2.96	17.10 ± 3.09	<0.001
T1 Carbohydrate Intake (%TE)	521	50.31 ± 6.65	50.32 ± 6.35	50.31 ± 6.47	0.996
T2 Carbohydrate Intake (%TE)	529	48.99 ± 5.91	49.88 ± 5.94	49.48 ± 5.94	0.089
T3 Carbohydrate Intake (%TE)	541	49.05 ± 5.50	49.96 ± 6.09	49.57 ± 5.85	0.072
T1Total Fat Intake (%TE)	521	35.47 ± 5.92	35.83 ± 5.45	35.68 ± 5.66	0.471
T2 Total Fat Intake (%TE)	529	36.11 ± 5.35	36.30 ± 5.43	36.22 ± 5.39	0.673
T3 Total Fat Intake (%TE)	541	36.19 ± 5.26	36.20 ± 5.40	36.19 ± 5.33	0.978
T1 SFA^3^ Intake (%TE)	521	13.35 ± 3.00	13.76 ± 2.93	13.57 ± 2.97	0.119
T2 SFA^3^ Intake (%TE)	529	13.45 ± 3.00	13.95 ± 3.00	13.72 ± 3.02	0.055
T3 SFA^3^ Intake (%TE)	541	14.00 ± 3.17	13.90 ± 3.10	13.93 ± 3.14	0.721
T1 MUFA^4^ Intake (%TE)	521	11.09 ± 2.55	11.38 ± 2.40	11.26 ± 2.47	0.184
T2 MUFA^4^ Intake (%TE)	529	11.41 ± 2.37	11.49 ± 2.26	11.46 ± 2.31	0.695
T3 MUFA^4^ Intake (%TE)	541	11.25 ± 2.29	11.38 ± 2.21	11.33 ± 2.24	0.497
T1 PUFA^5^ Intake (%TE)	521	5.99 ± 2.10	5.81 ± 2.11	5.90 ± 2.12	0.347
T2 PUFA^5^ Intake (%TE)	529	6.07 ± 1.76	5.78 ± 1.80	5.91 ± 1.80	0.066
T3 PUFA^5^ Intake (%TE)	541	5.79 ± 1.81	5.72 ± 1.87	5.76 ± 1.85	0.672
T1 Glycaemic Index	521	57.38 ± 4.24	57.71 ± 4.03	57.56 ± 4.12	0.365
T2 Glycaemic Index	529	56.26 ± 4.04	57.83 ± 3.71	57.13 ± 3.93	<0.001
T3 Glycaemic Index	541	56.12 ± 3.88	57.70 ± 3.88	57.00 ± 3.96	<0.001
T1 Glycaemic Load	521	132.40 ± 32.79	136.45 ± 38.70	134.69 ± 36.21	0.209
T2 Glycaemic Load	529	123.34 ± 31.42	140.26 ± 36.90	132.93 ± 35.62	<0.001
T3 Glycaemic Load	541	126.49 ± 30.03	139.81 ± 37.22	134.10 ± 34.96	<0.001
Neonatal Weight (kg)	542	4.05 ± 0.47	4.01 ± 0.45	4.02 ± 0.46	0.274
Neonatal Length (cm)	542	53.01 ± 2.40	52.48 ± 2.68	52.73 ± 2.56	0.160
Neonatal Abdominal Circ^6^ (cm)	222	33.24 ± 2.29	33.54 ± 1.96	33.40 ± 2.11	0.303
Neonatal Waist Circ^6^:Height Ratio	182	0.63 ± 0.04	0.64 ± 0.05	0.64 ± 0.05	0.013
Neonatal SOSF^7^ (mm)	186	28.66 ± 5.60	28.52 ± 4.88	28.58 ± 5.18	0.848
Neonatal Sum TSF^8^ and SSF^9^ (mm)	186	13.87 ± 2.83	13.92 ± 2.60	13.90 ± 2.69	0.906
Neonatal TSF^8^:SSF^9^ Ratio	186	1.01 ± 0.19	1.00 ± 0.18	1.00 ± 0.19	0.489
Neonatal Waist:Hip Circ^6^ Ratio	221	0.99 ± 0.06	1.00 ± 0.05	1.00 ± 0.06	0.192

“n” denotes number, %TE denotes percentage of total energy, p < 0.05 was considered statistically significant (2-tailed significance generated from ANOVA or t-tests as appropriate).

*1 hour post 50 g glucose challenge test.

Abbreviations: ^1^BMI, body mass index; ^2^ min, minutes; ^3^SFA, saturated fatty acids; ^4^MUFA, monounsaturated fatty acids; ^5^PUFA, polyunsaturated fatty acids; ^6^circ, circumference; ^7^SOSF, sum of skinfolds; ^8^TSF, triceps skinfold; ^9^SSF, subscapular skinfold.

### Underreporting

There was no difference in underreporting between the intervention and control groups (12.7% vs 9.7% definite underreporters respectively, p = 0.821). There was no difference in any of the associations in the final models when definite underreporters were removed with the exception of waist:length ratio, however the power of these models was reduced. Therefore the final models presented in Table 2. include underreporters. When definite underreporters were removed from the waist:length model, significant positive associations remained with maternal smoking and trimester 3 saturated fat intake while the associations with maternal age, mid-upper arm circumference, trimester 2 GL, postprandial glucose and group lost significance. In order to determine whether this was due to a lack of power or to underreporting being an actual confounder, underreporting was added to the final multiple linear regression model in order to control for it. Subsequently, all factors regained statistical significance with the exception of trimester 2 GL which originally had only showed a trend toward a significant association, and postprandial glucose which showed a trend (p = 0.050) towards a positive association with waist:length circumference when the model was adjusted for underreporting. This adjusted final model is presented in Table 2 in addition to the unadjusted model.

**Table 2:** **Maternal and paternal characteristics and maternal nutrient intakes associated with neonatal anthropometry – adjusted for maternal pre-pregnancy BMI, education level, length of gestation and neonate gender**^**1, 2**^

	**B**	**SEB**	**p-value**	**R**^**2**^**adj**	**F**	**p-value**
**Birth Weight**						
Gestational weight Gain	23.220	6.040	0.000	0.233	11.547	0.000
**Length**						
Mother baseline smoker	−6.220	1.490	0.000	0.278	6.193	0.000
**Abdominal Circumference**						
Trimester 3 SFA^3^ (%TE)	0.147	0.050	0.004	0.055	2.484	0.019
Trimester 3 PUFA^4^ (%TE)	−0.184	0.099	0.065			
**Thigh Circumference**						
Baseline strenuous physical activity	−0.706	0.257	0.014	0.467	4.365	0.008
**Chest Circumference**						
Maternal weight booking	0.150	0.080	0.080	0.377	2.906	0.039
Baseline strenuous physical activity	−0.820	0.286	0.012			
**Subscap skinfold thickness**						
Trimester 3 PUFA^4^ (%TE)	−0.140	0.074	0.062	0.050	2.301	0.038
**Subscapular:Triceps skinfold ratio**						
well-being% score	−0.003	0.001	0.006	0.051	2.410	0.030
**Waist Circumference:Length Ratio**						
Maternal age	−0.002	0.001	0.010			
Baseline mother smoker	0.083	0.018	0.000			
Baseline mother arm circumference	0.005	0.002	0.046			
Trimester 2 glycaemic load	0.003e^−1^	0.000	0.059	0.381	8.003	<0.001
Postprandial glucose at 28 weeks	0.007	0.003	0.036			
Trimester 3 SFA^3^ (%TE)	0.004	0.001	0.001			
Group	0.019	0.008	0.021			
**Waist Circumference:Length Ratio (adjusted for definite underreporting)**						
Maternal age	−0.002	0.001	0.008			
Baseline mother smoker	0.096	0.018	<0.001			
Baseline mother arm circumference	0.005	0.002	0.025	0.401	8.594	<0.001
Postprandial glucose at 28 weeks	0.006	0.003	0.050			
Trimester 3 SFA^3^ (%TE)	0.003	0.001	0.005			
Group	0.028	0.010	0.007			

^1^Well-established influences on neonatal size, ^2^Group affiliation was also included in all models as this study was a randomised control trial. %TE denotes percentage of total energy. Multiple regression analysis was used in this analysis and only statistically significant overall models were included. p < 0.05 was considered statistically significant.

Abbreviations: ^3^SFA, saturated fatty acids; ^4^PUFA, polyunsaturated fatty acids.

### Maternal and neonatal anthropometry

There was no difference in neonatal weight, length or other anthropometric measurements between the intervention and control groups except in thigh circumference measurement which has previously been described [44] and in neonatal waist:length ratio which was significantly lower in the intervention group. Mean maternal and neonatal anthropometric measurements are shown in Table 1. Maternal anthropometry was not significantly different at baseline but gestational weight gain was higher in the control group than the intervention group as described above.

Associations of maternal characteristics and macronutrient intake with neonatal anthropometry observed using simple linear regression are listed in Table 3. Statistically significant multiple linear regression models for the association between maternal characteristics and neonatal anthropometry are shown in Table 2. No significant multiple linear regression models existed for; hip circumference, triceps skinfold thickness, biceps skinfold thickness, thigh skinfold thickness, waist:hip circumference ratio, sum of subscapular and triceps skinfold thickness or for sum of all skinfold thicknesses. Statistically significant multiple regression models exist for birthweight, birthlength, abdominal circumference, thigh circumference, chest circumference, subscapular skinfold thickness, subscapular:triceps skinfold thickness ratio and waist circumference:length ratio as follows:

**Table 3:** Maternal and paternal characteristics and maternal nutrient intakes associated with neonatal anthropometry-unadjusted analysis

	**B**	**SE**	**p-value**	**R**^**2**^
**Birth Weight**				
Maternal weight booking (kg)	6.506	1.286	0.000	0.032
Maternal height (cm)	3.548	1.488	0.017	0.006
Paternal height (cm)	7.458	2.838	0.009	0.012
Maternal mid-upper arm circumference (cm)	18.656	5.223	0.000	0.016
Gestational weight gain (kg)	15.676	4.863	0.001	0.025
BMI^1^ booking (kg/m^2^)	11.752	3.717	0.002	0.012
**Birth Length**				
Paternal height (cm)	0.076	0.038	0.049	0.024
Attend gym (y/n)	−1.193	0.531	0.026	0.021
Mother baseline smoker (y/n)	2.724	0.964	0.005	0.031
Mother living with partner (y/n)	−1.350	0.507	0.008	0.027
Trimester 2 protein (%TE)	0.131	0.055	0.017	0.025
Trimester 2 carbohydrate (%TE)	−0.068	0.032	0.034	0.019
**Abdominal Circumference**				
Maternal weight booking (kg)	0.021	0.010	0.030	0.014
Trimester 1 SFA^2^ (%TE)	0.100	0.047	0.035	0.015
Trimester 3 SFA^2^ (%TE)	0.118	0.042	0.006	0.030
Trimester 3 PUFA^3^ (%TE)	−0.215	0.080	0.008	0.027
**Thigh Circumference**				
Group (intervention vs control)	0.404	0.200	0.044	0.012
Baseline strenuous physical activity (freq/wk)	−0.851	0.295	0.008	0.220
**Chest Circumference**				
Maternal weight booking (kg)	0.025	0.012	0.036	0.013
Baseline strenuous physical activity (freq/wk)	−0.882	0.356	0.020	0.166
Baseline attend gym (y/n)	−1.170	0.505	0.021	0.019
**Hip Circumference**				
Maternal weight booking (kg)	0.030	0.010	0.004	0.028
Maternal mid-upper arm circ^4^ booking (cm)	0.125	0.043	0.004	0.029
Maternal BMI^1^ booking (kg/m^2^)	0.082	0.030	0.007	0.024
**Mid-Upper Arm Circumference**				
Baseline strenuous physical activity (freq/wk)	−0.511	0.226	0.033	0.137
Trimester 2 protein (%TE)	−0.060	0.030	0.046	0.013
**Subscapular skinfold thickness**				
Trimester 3 PUFA^3^ (%TE)	−0.163	0.062	0.009	0.030
**Triceps skinfold thickness**				
Trimester 1 Glycaemic Index	−0.067	0.032	0.037	0.018
Trimester 3 PUFA^3^ (%TE)	−0.142	0.064	0.027	0.021
**Biceps skinfold thickness**				
Living with partner (y/n)	0.569	0.284	0.046	0.014
Trimester 3 PUFA^3^ (%TE)	−0.125	0.061	0.042	0.017
**Sum of All skinfold thicknesses**				
Trimester 3 PUFA^3^ (%TE)	−0.512	0.217	0.019	0.024
**Waist:Hip Circumference Ratio**				
Baseline mild physical activity (freq/wk)	−0.004	0.002	0.038	0.018
Timester 2 SFA^2^ (%TE)	0.003	0.001	0.028	0.017
**Sum of Triceps and Subscapular skinfold thickness**				
Trimester 3 PUFA^3^ (%TE)	−0.305	0.112	0.007	0.033
**Subscapular:Triceps skinfold thickness ratio**				
Percentage mood score	−0.003	0.001	0.010	0.029
**Waist Circumference:Length ratio**				
Maternal age (yrs)	−0.002	0.001	0.023	0.022
Baseline strenuous physical activity (freq/wk)	−0.019	0.007	0.013	0.232
Baseline smoker (y/n)	−0.072	0.017	0.000	0.070
Maternal mid-upper arm circ^4^ booking (cm)	0.002	0.001	0.031	0.018
Trimester 2 glycaemic load	0.000	0.000	0.005	0.039
Postprandial glucose at 28 weeks (mmol/l)	0.004	0.002	0.018	0.021
Trimester 2% energy from protein (%TE)	−0.002	0.001	0.016	0.026
Trimester 2% energy from SFA^2^ (%TE)	0.002	0.001	0.050	0.016
Trimester 3 SFA^2^ (%TE)	0.003	0.001	0.006	0.035
Trimester 3 PUFA^2^ (%TE)	−0.005	0.002	0.020	0.024

“n” denotes number, %TE denotes percentage of total energy. Simple Linear Regression was used in this analysis and only statistically significant associations were included in this table. p < 0.05 was considered statistically significant. There were no statistically significant associations with neonatal thigh skinfold thickness, therefore this variable is not included in the above table.

Abbreviations: ^1^BMI, body mass index; ^2^SFA, saturated fatty acids; ^3^PUFA, polyunsaturated fatty acids; ^4^circ, circumference.

Birthweight was positively associated with gestational weight gain R^2^_adj_23.3% (F = 11.547, p < 0.001). Birthlength was negatively associated with maternal smoking R^2^_adj_27.8% (F = 6.193, p < 0.001). Neonatal abdominal circumference was positively associated with maternal saturated fatty acid (SFA) intake and showed a negative trend towards association with polyunsaturated fatty acid (PUFA) intake in trimester 3 R^2^_adj_5.5% (F = 2.484, p = 0.019). Neonatal thigh circumference was negatively associated with frequency of strenuous physical activity reported in early pregnancy R^2^_adj_46.7% (F = 4.365, p = 0.008). Neonatal chest circumference showed a trend towards a positive association with maternal weight at booking and was significantly negatively associated with frequency of strenuous physical activity reported in early pregnancy R^2^_adj_37.7% (F = 2.906, p = 0.039). Neonatal subscapular skinfold thickness was showed a trend towards a negative association with PUFA intake in trimester 3 R^2^_adj_5.0% (F = 2.301, p = 0.038). Neonatal subscapular:triceps skinfold thickness ratio, a measure of central adiposity, was significantly negatively associated with percentage well-being score R^2^_adj_5.1% (F = 2.410, p = 0.030). Neonatal waist circumference:length ratio, another measure of central adiposity, was significantly negatively associated with maternal age and positively associated with maternal smoking, maternal mid-upper arm circumference (MUAC) in early pregnancy, SFA intake in trimester 3, postprandial glucose at 28 weeks gestation and membership of the control group and showed a trend towards a positive association with Glycaemic Load in trimester 2 R^2^_adj_38.1% (F = 8.003, p < 0.001).

### Macronutrient intakes

Maternal macronutrient intakes are displayed in Table 1. Their association with neonatal anthropometric measurements using simple linear regression are displayed in Table 3 and those that remained significantly associated with neonatal anthropometry when analysed using multiple linear regression are displayed in Table 2. SFA intake in trimester 3 was positively associated with neonatal abdominal circumference (B = 0.147, p = 0.004) while there was a trend towards PUFA intake in trimester 3 being negatively associated (B = −0.184, p = 0.065). There was a trend towards PUFA intake in trimester 3 being negatively associated with subscapular skinfold thickness (B = −0.140, p = 0.062). Finally, SFA intake in trimester 3 was positively associated with abdominal adiposity as measured by waist circumference:length ratio while there was a trend towards a positive association with GL in trimester 2 (B = 0.004, p = 0.001 and B = 0.003e^−1^, p = 0.059 respectively). There was no difference in maternal macronutrient intake between the control and intervention groups except for maternal GI and GL intake and protein intake as a percentage of total energy intake as previously described [27]. No significant association was found between level of concordance with a DASH diet and any of the neonatal body measurements examined. Similarly, previously defined healthy and unhealthy diet clusters were not associated with neonatal body measurements.

## Discussion

The main maternal factor associated with increased birth weight was greater gestational weight gain while the main maternal factor associated with greater birth length was non-smoking status. Neonatal central adiposity, determined using waist:length ratio, was negatively associated with maternal age, and positively associated with maternal smoking status, pre-pregnancy mid-upper arm circumference, trimester 3 saturated fat intake, postprandial glucose at 28 weeks gestation and membership of the control group and showed a trend towards a positive association with trimester 2 glycaemic load.

Similar to other studies [45-47], gestational weight gain was found to be positively associated with birthweight in this cohort. Guidelines from the Institute of Medicine (IOM) rely on pre-pregnancy BMI to determine an appropriate range of gestational weight gain [48]. It is well established that those who exceed the IOM guidelines are at risk of delivering a macrosomic infant [49]. Excess gestational weight gain increases the normal insulin resistance that occurs in pregnancy and may also affect other hormones that regulate nutrient transport across the placenta resulting in increased fetal insulin secretion, growth and adiposity [45]. Research by Ludwig et al. into multiparous women throughout successive pregnancies has found that gestational weight gain is responsible for increased birthweight despite controlling for genetic and sociodemographic factors [45].

Our finding that maternal smoking during pregnancy was associated with decreased birth length is well established in the literature [50,51]. Maternal smoking was also found to be positively associated with waist:length ratio, the equivalent of which (waist:height ratio) has been found to be a good measure of central adiposity in adults and children with a ratio of ≥0.5 indicating excess central adiposity [52]. A recent study by Brambilla et al. found it to be a better measure of adiposity than waist circumference or BMI in children and adolescents [53]. A meta-analysis by Ino [54] found that maternal smoking is associated with childhood overweight and obesity, possibly through a combination of the thrifty phenotype and catch-up growth during early infancy. Although maternal smoking during pregnancy is associated with reduced birth weight, length and relatively unchanged ponderal index in the literature [55,56], we were unable to identify any studies that had measured waist:length ratio at birth in relation to maternal smoking status. The only studies identified that had measured waist:length ratio at birth were from the same group and involved creation of normative waist:length centile charts at birth [57]. The increased waist:length ratio observed in the off-spring of smokers in this study appears to reflect the reduced height also observed and likely indicates that, while birthweight is not increased, central and visceral adiposity may be, increasing the risk of metabolic syndrome in later life.

Maternal early pregnancy MUAC was positively associated with neonatal waist:length ratio. MUAC has been found to be well correlated with maternal weight and BMI and remains stable in pregnancy i.e. unaffected by length of gestation [58]. As a measure of maternal overweight and obesity, MUAC has been found to be positively associated with birthweight [59-61]. Again, there is no information on its association with neonatal waist:length ratio to date to the best of our knowledge.

Trimester 3 SFA intake was positively associated with abdominal circumference and waist:length ratio. Trimester 3 PUFA intake showed a trend towards a negative association with abdominal circumference and subscapular skinfold thickness. There is a paucity of similar data into the effect of maternal fat intake in pregnancy on neonatal adiposity. High fat isocaloric diet in rats has been found to result in no difference in birthweight of pups [62]. Similarly, Brion et al. [63] found no association between maternal diet and child adiposity at 9 or 11 years of age in humans. However, the quality of dietary macronutrient intakes may be more important than absolute intakes and high SFA diet in pregnancy has been found by Murrin et al. [64] to be positively associated with child weight at age 5. Maternal intake of trans fatty acids in trimester 2 of pregnancy was also found by one group to be positively associated with birthweight [65] while other studies have reported conflicting results possibly due to examination of different trans fatty acids [17,66,67]. Fetal fat deposition increases with gestational age, therefore our observation that maternal fatty acid intake in trimester 3 is associated with neonatal adiposity is reasonable [3]. Although there is little research into the effect of SFA in pregnancy there is more interest into the effect of maternal dietary PUFA intake, in particular the possible anti-obesogenic effect of a greater omega 3:omega 6 ratio. However, a recent review by Hauner et al. [68] found that there is little evidence of this to date due to conflicting study results. Results of a prospective intervention study involving omega 3 supplementation and dietary reduction of omega 6 showed no effect on fat mass at age 1 [69]. This study, the INFAT study, similar to others involving fish oil supplementation found that birthweight and length of gestation were increased, however adiposity was not affected [69]. We believe ours is the first study to show an association between neonatal central adiposity and dietary fat quality.

Trimester 2 Glycaemic load (GL) was included in the multiple linear regression model and showed a trend towards a positive association with waist:length ratio. However, while the overall model, which included trimester 2 GL, was statistically significantly associated with waist:length ratio, T2 GL was not independently significantly associated and lost significance when underreporting was controlled for. Maternal postprandial blood glucose and membership of the control group were significantly positively associated with waist:length ratio although the relationship between postprandial glucose and waist:length circumference was reduced to a trend (p= 0.050) when underreporting was controlled for. There has been much research into glycaemic control and into the effect of dietary glycaemic index and load during pregnancy on birthweight due to the well-established risk of macrosomia in gestational diabetes [70]. The risk of macrosomia and increased neonatal adiposity has also been found to be increased towards the upper limits of normal blood glucose control in pregnancy [9,71,72] which is in line with our results. Low glycaemic index diet has been found to ameliorate the normal pregnancy-related increase in glucose intolerance associated with pregnancy resulting in fewer peaks in maternal postprandial glucose concentration [9]. The positive trend towards association between postprandial glucose and waist:length ratio in this study may indicate that the reduction in postprandial blood glucose levels observed with low glycaemic index diet was associated with reduced glucose transfer to the fetus and therefore less deposition of fetal adiposity. While the ROLO study found that the intervention group reduced the GI and GL of their diet, even when underreporters were excluded from the analysis [27], and had reduced glucose intolerance, no difference in birthweight was observed. In contrast, a recent retrospective analysis of members of the Danish National Birth Cohort has found that those in the highest GL quintile had significantly higher offspring birthweight than those in the lowest quintile [73] indicating that effects may be observed at extremes. The finding by the current study, that neonatal waist:length ratio was lower in the intervention group indicates that improved dietary carbohydrate quality may be associated with reduced central adiposity rather than birthweight at less extreme levels as the reduction in GI and GL observed in this study was quite modest.

This was a large, well-powered clinical trial designed to examine the effect of diet and lifestyle on neonatal size and adiposity. Rich dietary data was available at each trimester in combination with biochemical measures of glycaemic control. One limitation of this study was that detailed neonatal anthropometric data was not available for the full cohort, however weight and length measurements were taken for all infants. It should also be noted that this was a cohort at risk of macrosomia, therefore care should be taken regarding generalisation of results to other populations. A further limitation was that fatty acid composition was not broken down into omega 3, omega 6 and trans fats. This clinical trial was originally focused on the effect of GI, GL and glycaemic control on birthweight but nevertheless, data on SFA, MUFA, PUFA and total fat was available and this is the first human study to report an association between dietary fat quality and neonatal central adiposity.

## Conclusion

Several maternal diet and lifestyle factors were associated with neonatal body composition. The finding that neonatal central adiposity was positively associated with maternal dietary saturated fat and showed a negative trend with polyunsaturated fat and a significant negative association with membership of the low GI intervention group highlights the importance of dietary quality in pregnancy and the need for further research and education in this area.

## Competing interests

The authors declare that they have no competing interests.

## Authors’ contributions

MKH- carried out data analysis, assigned the DASH diet score and wrote the manuscript, CAMcG- carried out data entry and k-means cluster analysis, ERG gave advice on study design and editing of the manuscript, JMD carried out manuscript editing, FMMcA was responsible for conception of the study, advice on study design and manuscript editing. All authors read and approved the final manuscript.
